# Circadian Genes as Therapeutic Targets in Pancreatic Cancer

**DOI:** 10.3389/fendo.2020.00638

**Published:** 2020-09-11

**Authors:** María García-Costela, Julia Escudero-Feliú, Jose D. Puentes-Pardo, Sara Moreno San Juán, Sonia Morales-Santana, Sandra Ríos-Arrabal, Ángel Carazo, Josefa León

**Affiliations:** ^1^Research Unit, Biosanitary Research Institute of Granada, ibs.GRANADA, Granada, Spain; ^2^Cytometry and Michroscopy Research Service, Biosanitary Research Institute of Granada, ibs.GRANADA, Granada, Spain; ^3^Proteomic Research Service, Biosanitary Research Institute of Granada, ibs.GRANADA, Granada, Spain; ^4^Endocrinology Unit, Endocrinology Division, CIBER of Fragility and Healthy Aging (CIBERFES), San Cecilio University Hospital, Granada, Spain; ^5^Genomic Research Service, Biosanitary Research Institute of Granada, ibs.GRANADA, Granada, Spain; ^6^Clinical Management Unit of Digestive Disease, San Cecilio University Hospital, Granada, Spain

**Keywords:** pancreatic cancer, circadian clock, chemotherapy, metabolism, precision medicine

## Abstract

Pancreatic cancer is one of the most lethal cancers worldwide due to its symptoms, early metastasis, and chemoresistance. Thus, the mechanisms contributing to pancreatic cancer progression require further exploration. Circadian rhythms are the daily oscillations of multiple biological processes regulated by an endogenous clock. Several evidences suggest that the circadian clock may play an important role in the cell cycle, cell proliferation and apoptosis. In addition, timing of chemotherapy or radiation treatment can influence the efficacy and toxicity treatment. Here, we revisit the studies on circadian clock as an emerging target for therapy in pancreatic cancer. We highlight those potential circadian genes regulators that are commonly affected in pancreatic cancer according to most recent reports.

## Introduction

Pancreatic cancer is the 15th most common malignancy and ranks 7th among malignant cancers in terms of death (1). On top of that, this cancer represents one of the most malignant types of tumors, being the fourth leading cause of cancer-related death in the United States and the sixth in China (1). Unfortunately, without any substantive improvement in the current curative therapies, the pancreatic ductal adenocarcinoma could possibly be the second leading cause of cancer-related death by 2030 (2).

The mammalian circadian system consists of a central clock in the hypothalamic suprachiasmatic nucleus (SCN), and peripheral clocks in other brain regions and tissues throughout the body, including muscle, adipose tissue, liver, colon and pancreas. The SCN receives information about the 24 h light:dark cycle trough the retina and this timing signal is forwarded via neural and hormonal signals and body temperature to the peripheral clocks in order to maintain organism homeostasis (3, 4). This circadian clock is essential for the endocrine system, as it requires the accurate timing communication among the different organs that composed it to ensure the correct response and anticipation to the environmental changes or stresses (5). Disruption on circadian clock has been also linked to initiation, progression, metastasis, and resistance to treatments in cancer (6–11). Thus, analysis of circadian function could lead could lead to the identification of prognostic markers and therapeutic targets for specific cancers (12).

Specifically, the pancreas is one of the organs most influenced by this timing system, since it possesses its own clock regulated by the SCN and non-photic signals to keep its normal physiology; but also because it is closely linked to its endocrine function (13). All this knowledge has led to the development of clinical trials to improve its function trough time management of treatments (14, 15). *In vitro* and *in vivo* studies demonstrated that circadian clock disruption is also linked to the development and progression as well as treatments resistance in pancreatic cancer. In this review, we highlight most recent results in such aspects of this type of cancer that could help the identification of prognostic and predictive factors and the development of personalized medicine for patients suffering from this disease.

## The Molecular Circadian Clock

The rotation of the Earth around its own axis each 24 h generates a constant daily sunlight-darkness cycle, which conditions the behavior and the activity of organisms. In order to adapt and anticipate to the environmental cues resulting from these 24-h rhythmic oscillations, organisms have developed an endogenous timing-keeping mechanism known as circadian clock. However, this circadian clock regulates rhythmicity far beyond sleep-wake cycles. Circadian clock governs physiological and biochemical rhythms of processes related to hormone secretion, blood pressure, cell division, body temperature, immune function and metabolism, since they fluctuate over an ~24-h period (16). These rhythms have three characteristic hallmarks: they drive rhythms with a period of about 24-h, which are endogenous and persist in constant conditions; they remain synchronized with their environment, being resettable by external changes; and they should be temperature compensated, i.e., their periods are not affected by temperature (17). Disruption in functional circadian rhythm results in different physiological disorders such as cardiovascular diseases (18), metabolic syndrome (16), neurodegenerative disease (19), endocrine disruption and cancer (20).

In mammals, the circadian system is organized in a hierarchical system composed by a network of multi-oscillator circadian clocks. This network integrates a *master pacemaker* or central clock, which is located in the hypothalamic SCN, and a series of peripheral clocks present in virtually all tissues and cells. The SCN is able to receive information of the external light through the retina to couple the daily physiological rhythms to the environmental cues. This information is transmitted via neural and humoral pathways to the peripheral clocks in order to synchronize them and to provide proper rhythms in these tissues (21). However, light is not the only signal that allows peripheral clocks to entrain the rhythms, since they could response to non-photonic signals such as food (22) or drugs (23). Peripheral clocks can also generate circadian rhythms independently, being observed in cultured cells (24), or even in stem cells upon differentiation during development (25, 26).

At molecular level, circadian rhythms are controlled by a cell-autonomous genetic network of transcription/translational feedback loops (TTFL) of circadian clock genes that regulate themselves. The core loop is driven by the expression of *BMAL1* (Brain and Muscle ARNT-Like 1) and *CLOCK* (Circadian Locomotor Output Cycles Kaput), which are expressed during the day, interacting with each other to produce CLOCK:BMAL1 heterodimers in the cytoplasm. These heterodimers are translocated to the nucleus, where they act as transcription factors of elements containing E-boxes. Their targets include the transcriptional repressors period (*PER1-3*) and cryptochrome (*CRY1-2*) genes. PER and CRY proteins can also form a heterodimer in the cytosol. Once translocated to the nucleus, PER:CRY heterodimers binds to CLOCK:BMAL1, inhibiting their own transcription and decreasing PER and CRY protein levels. This allows a new cycle of transcription by CLOCK:BMAL1, closing the cycle. Additionally, BMAL1 levels are also tuned by a stabilizing loop controlled by *ROR*α and *REV-ER*α*-*β, whose expression is controlled by CLOCK:BMAL1 as their promoters contain E-boxes. REV-ERα-β and RORα shared DNA binding motifs, competing between them to inhibit or promote, respectively, *BMAL1* expression (27, 28). CLOCK:BMAL1 also drives a transcriptional loop in which the factors DBP (D-box binding protein), TEF (thyrotroph embryonic factor), and HLF (hepatic leukemia factor) interacts at sites containing D-boxes with the repressor NFIL3 (Nuclear Factor, Interleukin 3 Regulated; or E4BP4), which is driven by the REV-ERB/ROR loop (29, 30). Proteins of the core circadian clock drive rhythmic expression of the called clock-controlled genes (CCGs). These CCGs are tissue-specific, and ultimately are responsible for the physiological requirements and characteristics of every organ, accounting for 43% of all protein-coding genes in at least one organ (31).

The molecular regulation of the circadian clock is far beyond of just TTFLs. Recently, it has been described an epigenetic control of circadian transcription, at the level of DNA methylation as well as in the modifications of histones. Additionally, tissue-specific transcription factors, nuclear receptors, or some regulators of intracellular signaling can determine a portion of the circadian transcriptome in response to environmental cues (32, 33).

In addition, a machinery of post-translational modifications is involved in the regulation of the correct ticking of the clock, adding a new layer of complexity (34). Phosphorylation is one of the key post-translational modifications, which is involved in regulating the core clock loop, as well as the downstream CCGs (35). The stability/degradation rate of the PER and CRY proteins is mediated by different pathways and is a key issue to maintaining the rhythm period. CK1ε/δ-mediated phosphorylation of PER proteins is necessary for their ubiquitination by βTrCP and degradation by the 26S proteasome. CRY1 and CRY2 are phosphorylated by AMPK and DYRK1A/GSK-3β, respectively, and then polyubiquitinated by FBXL3 (35). Acetylation/deacetylation is another mechanism of post-translational modifications of clock proteins. Although the enzyme(s) responsible for PER2 acetylation remain(s) unknown, Asher et al. reported that SIRT1, a class III histone deacetylase, is bound to the CLOCK:BMAL1 complex in a circadian fashion and promotes the deacetylation and subsequent degradation of PER2 (36). Nakahata et al. observed that SIRT1 is bound to the CLOCK:BMAL1 complex at the circadian promoters and deacetylates BMAL1 at Lys537 (37). Furthermore, the inhibition of SIRT1 activity leads to significant disturbances in the circadian cycle and to the acetylation of histone 3 (H3) and BMAL1 (36). Interestingly, the HDAC activity of SIRT1 is regulated in a circadian manner both *in vitro* and *in vivo* (36). Further post-translational modifications that participate in tuning the molecular clock and clock-controlled proteins comprise ubiquitination, SUMOylation, or methylation (38).

Embryonic stem cells express circadian clock proteins, but they do not oscillate as they do not possess a functional TTFL (25, 26), however they show circadian rhythms for glucose utilization and glucose transporter transcription. This reveals the existence of circadian rhythms independent of the molecular clock (39). The main evidence of transcriptional-independent circadian rhythm comes from red blood cells (RBCs), since they are anucleated cells, and therefore incapable of transcription. RBCs show circadian peroxiredoxins (PRDX) oxidation/reduction rhythms (40). PRDX are a family of conserved enzymes involved in the regulation of peroxide levels. A subclass of PRDX, known as 2-Cys PRDX, during the process of reducing peroxide is oxidized to a sulfenic acid form (PRX-SO), which need to be reduced by a thioredoxin to be active again. Nevertheless, a fraction of PRX-SO is hyperoxidized to sulphinic and sulphonic acid forms (PRX-SO2 and PRX-SO3), which gives rise to an inactive form of the enzyme until they are recycled by a sulfiredoxin through an ATP-dependent reduction (41, 42). The levels of these forms oscillate through a 24-h pattern and met the 3 hallmarks of circadian rhythms previously indicated. RBCs also show circadian oscillations in others redox processes such as hemoglobin tetramer-dimer transition and NAD(P)^+^/NAD(P)H oscillations (40). The TTFLs and redox/metabolic cycles are interconnected and individually necessary for the maintenance of rhythms at the cellular level (43–46). The existence of non-transcriptional clock mechanisms along with the evidences of interplay between cellular redox/metabolic oscillations and circadian clocks (47) suggested a new layer in the circadian system. While regulation of metabolic output by the circadian clock is well-established, the mechanism by which metabolite fluctuations can also contribute to circadian clock adjustments is less know (48–50). Very recently, Levine et al. demonstrated in aged mice a role for NAD+ in remodeling the circadian transcriptome through SIRT1-mediated deacetylation of the core clock repressor PER2, which in turn promotes the activity of CLOCK/BMAL1 (51). Other studies revealed rapamycin (TOR) kinase, a conserved central growth regulator, as essential for the metabolic control of the circadian period in plants (52). In mammals, the tight junction protein 1 (TJP1) functions as a mediator of mTOR to modulate the hepatic circadian clock (53). Thus, perturbations in the circadian clocks are a hallmark of human metabolic diseases (54).

## Circadian Rhythms and Cancer

The closely interplay between the circadian system and cancer has been shown through epidemiological studies, in which associations between shift work and higher risk for developing cancer has been found (8, 10, 55). Human rotating working schedules (as cause for circadian disruption) are, in fact, considered by the WHO as a IARC 2A carcinogen (56); but it is not the only situation related to schedule that can contribute to cancer. For instance, a Spanish study found a relationship between having dinner before 9 p.m., and long interval between last meal and sleep, with a lower risk for breast and prostate cancer (9). Additionally, loss of circadian homeostasis is associated with poor prognosis, worse response to treatment and higher mortality in different cancers (6, 7).

These evidences have been corroborated in animal model studies, in which genetic and environmental controlled circadian disruption promotes cancer development. Filipski et al. showed how Glasgow Sarcoma progression was accelerated in mice under chronic jet lag conditions (an advance of light onset by 8 h every 2 days) rather those exposed to control conditions (alternation of 12 h of light and 12 h of darkness, normal circadian rhythmicity) (57). A similar effect was seen by Papagiannakopoulos et al., who saw decreased survival and promoted lung tumor growth and progression in mice exposed to prolonged jet lag conditions (58). In the same study, the authors genetically manipulated the circadian genes *Per2* (*Per2*^m/m^) and *Bmal1* (*Bmal1*^−/−^) in whole-animal bodies, exhibiting the same previous effects describes, and so, pointing out their tumor-suppressive role in lung cancer (58).

The contribution of a gene to cancer development is established in base of its observed mutation rate. Although clock genes mutations occur at low rate, they are related with prognosis and overall survival in cancer. In addition, their expression seems to be altered in several cancers (59). Ye et al., systematically analyzed the genetic and clinical data of The Cancer Genome Atlas (TCGA) in relation to clock genes (core and related clock genes) for 14 cancer types, showing that 90.2% of these genes were differentially expressed in at least one cancer type (59). *BMAL2* and *REV-ER*α were upregulated in 9 and 8 cancer types, respectively, suggesting they may act as oncogenes. Meanwhile, others clock genes were downregulated including *PER1* in 11 cancer types, *PER2* in 7, *PER3* and *CRY2* in 10 and *ROR*α in 11, indicating they may act as tumor suppressor genes (59). However, some of them despite of the overall downregulated expression were found upregulated in few cancer types. In this context, *CRY2* and *PER3* showed overall downregulation but were upregulated in kidney chromophobe and in kidney renal clear cell carcinoma (KIRC), respectively; or *NR1D1*, which was upregulated in general, was downregulated in BRCA triple-negative breast cancer (59). These data suggest that clock genes may play different roles in different tumor environments and tissues. Clock genes are highly associated with activation or inhibition of oncogenic pathways, with *CRY2* playing a dominant role in cancer development (59).

Circadian genes, along with the circadian rhythm itself, may participate in the development of cancer hallmarks: deregulation of cell cycle, apoptosis, DNA damage response and metabolism. The cell cycle depends on the building up complex of specific cyclin-dependent kinases (CDKs) and cyclins at specific times in a very tight process in order to restrict uncontrolled proliferation. Despite of the similarities about the oscillatory behavior between the cell cycle and circadian clock, its relationship is still not fully understood. Clock proteins control the circadian expression of several cell cycle components such as Wee1, c-Myc, p20, p21, and p53 among others (60). WEE1 protein transcription, an inhibitor of G2-M transition, is activated by BMAL1:CLOCK, but, inhibited by PER and CRY allowing the cell cycle to continue (61). C-MYC plays a pivotal role in cancer taking part in multiple processes, such as metabolism, cell cycle and apoptosis, and its deregulation along with the loss of p53 is associated with the maintenance of the cancer stem cells (CSC) pool as well as the promotion of genomic instability and the inhibition of apoptosis even after severe damage (62, 63). C-myc gene is directly suppressed by CLOCK:BMAL1 since its promoter possess E-boxes (64), and is stabilized by PER1, suppressing p21 expression, allowing the cell cycle to continue (65). P21, is also inhibited or activated by REV-ERBα and RORα, respectively (66). At protein level, c-MYC turnover is regulated by CRY2. In this process, CRY2 cooperates with the E3 substrate receptor FBXL3 to degrade c-MYC in a phospho-specific manner contributing to circadian protection from tumorigenesis (67). P53 is a transcription factor involved in several biological processes, whose products exert a tight control of many cell cycle checkpoints. P53 stability and transcriptional activity is modulated by PER2 in unstressed cells by blocking Mdm2-dependent ubiquitination and transcription of p53 target genes (68). Per2 forms a trimeric complex with p53 and Mdm2 (68) and dimeric complexes with p53 alone with differential spatial distribution that also influences p53's downstream response under genotoxic stress (69). However, the phase relationship between PER2 and p53 are opposite, revealing a node of regulation where circadian and checkpoint components can bi-directionally communicate and influence each other's downstream signaling (70). In fact, PER2 interact with MDM2, and MDM2 target of PER2 for degradation (71). In addition, p53 also regulates PER levels by binding to its promoter and repressing it, resulting in a disruption of the positive loop and shifting of circadian cycle (72).

Another link between the circadian clock and the cell cycle is exerted through the response to DNA damage mediated by ATM and ATR. In humans, the circadian protein Timeless (TIM) exerts the link among cell cycle, DNA damage response and circadian rhythm. Human TIM was found to form a complex with CRY2, checkpoint kinase 1 (CHK1) and ATR to regulate S phase and DNA damage response (73). This is not the only cell cycle checkpoint in which TIM is involved, as it also interacts with CRY to regulate the ATR/CHK1-mediated G1/S transition checkpoint, as well as the G2-M transition by interacting with ATM/checkpoint kinase 2 (CHK2) and PER (65, 74).

Regarding cancer cells metabolism, tumor cells reprogram their metabolism toward a high glycolysis rate followed to lactate fermentation even at oxygen conditions (75). This increase of glycolysis leads to an increment of reactive oxygen species (ROS), and the subsequent DNA damage; accumulation of intermediates rerouted to biosynthetic pathways for growing and proliferation (75); and the acidification of the microenvironment, which in turn may disrupt the circadian clock at the niche (76). The metabolism programme switch may be produced by the activation of PI3K/AKT pathway, and by the hypoxia response pathway mediated by HIF-1α, both related with circadian rhythm (77, 78). Specifically, HIF-1α was found to be a direct transcriptional target of NPAS2, which mediates both the upregulation of glycolytic genes and the downregulation of mitochondrial biogenesis in hepatocellular carcinoma (HCC) (79). It should be noted that, in this case, overexpression of a component of the circadian clock occurs, instead of down-expression. On the contrary, lack of PER2 increases PI3K/AKT/mTOR signaling pathway, protein synthesis and cell proliferation and decreases autophagy in this type of cancer (80). Other signaling pathways implicated in HCC development are regulated trough circadian clock, such as WNT/β-Catenin, MAP Kinase and Hedgehog signaling pathways. Other studies showed that loss of hepatocyte nuclear factor 4 alpha (HNF4α) in the liver combined with high fat feeding in mice induced EMT in an IL6-dependent manner (81). The HNF4α gene encodes different isoforms. P1-HNF4α, the predominant isoform expressed in the adult liver, inhibits the expression of tumor promoting genes in a circadian manner. In contrast, P2-HNF4α isoform represses BMAL1, causes cytoplasmic re-localization of P1-HNF4α and HCC grown (82). Therefore, a deeper understanding of circadian clock is promising for developing new strategies for HCC prevention and management (83), a disease in growing incidence in both men and women, because it is linked to non-alcoholic fatty liver disease (NAFLD) and to obesity (81).

Translated to the clinical practice, all these processes controlled, to a greater or lesser extent, by the circadian clock are interesting as possible targets for new approaches. To the day, circadian rhythms are being used as a treatment in two different ways: targeting the clock or CCG products and chronotherapy. The first approach tries to target the circadian components or restores normal circadian oscillations. A recent study shows how SR9009 and SR9011, two agonists against REV-ERBs, impair glioblastoma growth and survival without affecting surrounding healthy cells in mice (84), although the authors did not analyze whether these compounds affect central or local (tumoral) circadian rhythmicity. Other authors have seen how treatments with dexamethasone, forskolin and heat shock to induce circadian rhythmicity slow down tumor growth on B16 murine melanoma cells and human colon carcinoma HCT116 (85).

On the other hand, chronotherapy, the administration of a drug at optimal times based on the individual's circadian rhythm in order to maximize the therapeutic effects and minimize the toxic ones, appears to be a new way to improve the current treatments. Li et al. developed a MAP Bayesian interference method to determine the optimal timing for irinotecan administration based on rREV-ERBα and BMAL1 expression, achieving a decrease of toxicity (86). Another study carried out by Dakup et al. points out the application of cisplatin, a widely used chemotherapeutic agent but limited by its toxicity and resistance, at the evening reduces toxicity compared to the morning in wild type mice, but no time-of-the day difference was observed in *Per1/2*^−/−^ (87). Since it is known that most of the drug targets, not only anti-cancer drugs, are components with circadian behavior (31), the optimum circadian time for their application should be determined before trial phases, as well as, it opens the door to reconsider drugs that were not successful in II and III trial phases, and applied them in a circadian manner. However, it is important to note that, its application to clinical practice is still complicated since circadian rhythms are not fully understood, due to the necessity for the optimization of drug protocols to be as efficient as possible and tumor heterogeneity.

Tumor heterogeneity is associated, in part, to the presence of CSCs (88). CSCs are a distinct proportion of the cancer cells present in the tumor, characterized by their tumorigenesis, differentiation and self-renewal capacities, but also by a slow cell cycle and an increase of ABC transporters which allow them to evade cancer therapies (89). Those characteristics have them led to be considered as a key player in metastasis and relapse, and may be the origin of a cancer process. The circadian properties of CSCs remain unknown, likely to the difficulty to isolated, based on the presence of stemness markers such as ALDH1, CD44, or CD133 among others, and work with them *in vitro* and *in vivo* (90). However, some interesting progresses has been made with the role of circadian clock in leukemic stem cells (LSCs) (91) and glioblastoma stem cells (GSCs) (92). Puram et al., using a murine model of acute myeloid leukemia (AML) and *in vitro* human cells lines, found that normal and malignant hematopoietic cells harbor an intact robust clock, but the knockout of *BMAL1* and *CLOCK* resulted in an impairment of tumor cells, meanwhile normal hematopoietic function was not affected. The knockout also results in an increment of myeloid differentiation markers and reduction of stem cell markers in leukemia cells, pointing out the importance of the circadian role in the maintenance and function of LSCs (91). On the other hand, Dong et al., showed how *BMAL1* and *CLOCK* act as oncogenes in glioblastoma. These genes are necessary for GSCs proliferation and survival, but they are not required for differentiated glioblastoma cells or normal neural stem cells. In GSCs, the clock machinery seems to regulate glucose metabolism and lipid synthesis, while this control is not exerted in normal neural stem cells, being these differences attributed to distinctive changes in chromatin (92). Both cases show the relevance of circadian programming in CSCs, and, although it is still poorly understood, they reflect how is the reprogramming of the circadian regulation, rather than the rhythm itself, what is needed to understand in CSCs and cancer in order to use the circadian clock as target or tool for therapies. Circadian dynamics of CSCs are regulated by the tumor microenvironmental factors. The number of CSCs (high ALDH activity cells) in a mouse breast tumor model exhibited accused circadian alterations, which was a cause of the time-dependent release of WNT10a from non-CSCs (ALDH-negative cells) of tumors. With these results in mind, Matsunaga et al., demonstrated that the antitumor and antimetastatic effects of the ALDH inhibitor N,N diethylaminobenzaldehyde (DEAB) were improved by changing the dosing schedule of the drug (93).

As mentioned before, circadian rhythms are regulated in part by epigenetics modifications; however the link between circadian clock and epigenetic machinery is reciprocal (32, 33, 94). Circadian gene silencing has been implicated in several aspects of cancer, directly as well as indirectly due to regulation of cancer-related CCGs (94). On the other hand, the circadian molecular system modulates the daily variation in DNA methylation, with increased levels at night (95). In relation to histone modification, core clock genes and clock output genes underlay rhythmic histone modifications. Core circadian genes are regulated by histone phosphorylation, acetylation and methylation (94). Interestingly, CLOCK protein has histone acetyltransferase (HAT) activity directed to BMAL1 and histone H3 (96) and, acting with other HAT, to the promoters of CCGs to enhance transcription (97). SIRT1 counteract the activity of CLOCK and deacetylates BMAL1, as described previously (36, 37). Other acetylases and histone deacetylases, participate in the regulation of clock gene transcription. Alterations in these epigenetic control mechanisms could be a key factor during carcinogenesis (32). In addition, aberrant patterns of histone methylation and phosphorylation have been reported in cancer (33).

## The Role of Clock Genes in Exocrine and Endocrine Pancreatic Function

Peripheral oscillators operating in metabolic organs, such as the liver, muscle, white adipose tissue, pancreas and gut, function in concert with the central clock to orchestrate glucose, lipid and protein metabolism (54). The pancreas is an organ with a dual role: endocrine and exocrine functions.

As exocrine gland, it secretes pancreatic juice with digestive enzymes into the duodenum. In this case, circadian clock is involved in two important aspects of pancreatic physiology. Throughout embryonic development, the core circadian gene Clock regulates timing of pancreatic differentiation in mouse through the Wnt and Notch pathways as well as cell cycle regulators. Disruption of Clock and Timeless in the embryonic pancreas does not block pancreatic differentiation but alters the balance and maturity of endocrine and exocrine cells (98). Exocrine pancreas in adult rats exhibits a chrono-morphological pattern in the shape coefficient of glandular acini (99).

Exocrine pancreatic secretion showed a circadian rhythm, with secretory rates increasing in the dark period and decreasing during the light period in rats (100). In pigs, a biphasic pattern of the exocrine pancreas, postprandial peak and between meals was detected (101). In humans, pancreatic exocrine secretion and intestinal motility are coupled with different circadian rhythms. Intestinal motility follows a circadian rhythm with reduced motor activity during the night. In contrast, pancreatic amylase output increases significantly during the night, whereas protease output remains unchanged. The cyclical coupling between interdigestive motility and pancreatic secretion is preserved throughout the circadian cycle and seems to be not caused by increased cholinergic activity (102). In the course of chronic pancreatitis, the presence of severe exocrine insufficiency induce longer periods of gastric acid exposure, indicating that gastric acidity, exocrine pancreatic insufficiency, and impaired digestion are closely related (103).

The endocrine function is exerted by the islets of Langerhans composed of α-cells and β-cells. Those cells are responsible for controlling glycaemia by secreting glucagon (α-cells) and insulin (β-cells) at hypoglycaemic or hyperglycemic conditions, respectively, with opposite effects on glucose metabolism (104). An impairment on insulin secretory function of β-cells as well as the increasing of β-cell apoptosis are the principal abnormalities which lead to insulin resistance in liver, muscle and adipose tissue; and eventually to the development of type 2 diabetes mellitus (T2DM) (105). It is widely accepted that the development of T2DM is caused by multiple factors combining lifestyle (obesity and diet mainly) and genetics (106). However, in the last few years, there is a growing evidence of the association between alterations in the circadian rhythms and the development of T2DM (107–109). In fact, some studies, in mice and humans, highlight the necessity of a functional circadian clock on the pancreatic isles cells for proper insulin secretion (50, 110).

Under physiological conditions, humans present a strong time-of-day effect in glucose tolerance, with a peak in the morning/awakening, concurring with the maximal glucose plasma concentrations and glucose uptake. This glucose tolerance is decreased through the day, being minimal at night, likely as an effect of insulin tolerance, showing a circadian behavior (111).

Briefly, the production and release of insulin from β-cells involve the production of insulin and granule formation; glucose uptake by GLUT2 receptors; and glycolysis and ATP formation by mitochondria, which initiates a cascade reaction mediated by inhibition of ATP-sensitive potassium channels and opening of voltage-dependent calcium channels. This increases Ca^2+^ intracellular levels which eventually results in glucose-stimulated insulin granule secretion. Almost every of these processes are impaired in T2DM, and are under the tight control of the circadian genes, as demonstrated in *BMAL1* and *CLOCK* knockout studies in different *in vitro* and *in vivo* models (110, 112, 113).

Saini et al., using small-interfering RNA-mediated knockdown of *CLOCK* (siClock) demonstrated the profound impact of *CLOCK* disruption on insulin secretion (110). The disruption of *CLOCK* gene does not affect Insulin (*INS*) expression but it downregulates the expression of *SLC30A8, VAMP3*, and *STX6* which are involved in insulin granule formation, vesicle trafficking, and secretion (110). *SLC30A8* encodes for the Zinc-Transporter SCL30A8 which is required for proper insulin synthesis, storage and secretion (114), meanwhile VAMP3 and STX6 proteins take part of a complex involve in granule fusion with the plasma membrane (115). *CLOCK* disruption also affects to the intracellular signal transduction cascade required for glucose-stimulated granule secretion, as *GNAQ* (Guanine nucleotide-binding protein, subunit α), *ATP52G2* (ATP synthase, mitochondrial F0 complex H^+^, subunit C2), and *ATP1A2* (Na+/K+-transporting ATPase, subunit α-1) are downregulated, and *KCNJ11* (potassium channel, inwardly rectifying subfamily J member 11) is upregulated, leading to decrease glucose-stimulated ATP production and membrane hyperpolarization, respectively, altering glucose-induced insulin secretion (110).

On the other hand, *Bmal1* disruption reveals the importance of Bmal1 in the prevention of oxidative stress in β-cells. Lee et al. generated mice with β-cell deleted *Bmal1* (β*-Bmal1*^−/−^), showing an increase of ROS and mitochondrial uncoupling (116). The absence of functional Bmal1 results in the decrease of the antioxidant regulatory factor Nrf2, and so the absence of its transcriptional target genes *Sestrin2, Prdx3, Gclm*, and *Gclc*, which inhibits the antioxidant responses in β*-Bmal1*^−/−^ (116). Parallel to these events, or as a result of the ROS accumulation increase, there is an upregulation of *Ucp2* which generates oxidative stress-induced uncoupling of mitochondria and impairment of glucose-induced insulin secretion (116).

The loss of β-cells by apoptosis, another feature of T2DM, can be linked to a non-functional circadian clock. Saini et al., reported a downregulation of genes that can reduce apoptotic cell death (*FURIN, PRDX3, PPP2CB*, and *BTG*) and an upregulation of genes that can induce apoptosis (*PSEN2, SQSTM1, ACVR1B, GCH1, HTT*, and *ARHGEF3*) after clock disruption (110). Also, the absence of Prdx3 and Gclc, two important agents in preventing oxidative stress-induced apoptosis in β-cells, in β*-Bmal1*^−/−^ may trigger the death of pancreatic β-cells (116, 117).

Another circadian gene that play an important role in endocrine pancreatic function is *REV-ERB*α whose lack of expression is related to impaired insulin secretion and cell proliferation reduction (118). Outside of the circadian genes, there are other genes with circadian-manner expression with have an important role on β-cells homeostasis such as *DBP* (albumin site d-binding protein) and *TEF* (Thyrotroph embryonic factor) transcription factor. TEF seems to control the expression of GLUT2 under circadian behavior (119), meanwhile DBP regulates the circadian expression of *ARNT/HIF-1*β, which may have a role in connecting circadian rhythm and metabolism, being impaired in T2DM (120). Taken together, the endocrine pancreatic function is strongly associated with circadian rhythms. Alteration of the rhythms leads to an impairment function of β-cells and increasing risks for of T2DM development.

Non-alcoholic fatty pancreas disease (NAFPD) describes a phenotype of pancreatic steatosis not caused by alcohol consumption, viral infections, toxins, or congenital metabolic syndromes, and ranging from deposition of fat in the pancreas to pancreatic inflammation, and resultant fibrosis. Together with T2DM, cardiovascular and cerebrovascular diseases and non-alcoholic fatty liver disease (NAFLD) is considered a pathology associated with the metabolic syndrome (121). NAFPD is also considered as a risk factor for pancreatic adenocarcinoma (122). Although NAFPD and NAFLD have different natural history and pathophysiology, both diseases are associated to obesity. In fact, in an obesity mouse model, the combination of maternal obesity (MO) and obesogenic diets predisposes offspring to obesity and to NAFPD through mechanisms involving perturbations in core circadian genes expression (123). In this model, offspring fed obesogenic diets also developed NAFLD phenotype in which hepatic rhythmicity of endoplasmic reticulum stress is disrupted (124).

## Epidemiology and Risk Factors in Pancreatic Cancer

The most common type of pancreatic cancer, adenocarcinoma of the pancreas, starts with an uncontrolled growth of exocrine cells (forming glands and ducts) in the pancreas (125). About 95% of pancreatic cancers are adenocarcinomas, usually starting in the pancreatic ducts. There are other types of exocrine pancreatic cancers which are less common, as adenosquamous carcinomas, squamous cell carcinomas or ampullary cancer, and also endocrine pancreatic cancers, in a very low percentage (125).

Each type of pancreatic cancer can present different characteristics and possible therapies; we will focus on the epidemiology and risk factors of the predominant malignant pancreatic cancer, the ductal adenocarcinoma. This is a highly lethal malignancy, causing more than 45,000 deaths in the United States each year, among <60 000 patients diagnosed, which represents a very high death rate (126). According to the latest statistics of the American Cancer Society (page)^1^, only 9% of the patients will survive after 5 years since the diagnosis of the disease. An explanation behind this is that the pancreatic cancer presents non-specific symptoms, which lead to a delayed diagnosis and dismal prognosis (127). Actual therapies against pancreatic ductal adenocarcinoma include surgery, adjuvant chemotherapy, radiation therapy (intraoperative, in which a large dose of radiation is given to the area at the same time as the surgery, or the proton beam radiation) and targeted therapies (128).

The use of the different therapies against pancreatic ductal adenocarcinoma depends on the diagnosis of the tumor extension or staging. This is essential in order to determine tumor respectability, which represents the only potentially curative treatment for this cancer. The TMN (Tumor-Node-Metastasis) classification, established by the American Joint Committee on Cancer, is the most commonly used system for staging pancreatic ductal adenocarcinoma into four different stages: stages I or II (localized, resectable tumor), stage III (locally advanced tumor, unresectable) and stage IV (metastatic tumor, unresectable). Only 5% of tumors are classified as a borderline or potentially resectable tumor (129).

For localized tumors (stages I and II), surgical resection is the only potentially curative treatment. The most common surgical technique for pancreatic tumors is cephalic duodeno-pancreatectomy with or without pyloric preservation (127). Some groups recommend chemo-radiotherapy as an adjuvant treatment (130). However, other studies, including a very large number of patients, show that only adjuvant chemotherapy significantly improves survival and that chemo-radioterapy can even be harmful (131). The chemotherapeutic agents used in these cases are 5-fluoracil and gemcitabine (GEM) (127). For the localized advanced tumors, the treatment is usually chemotherapy with GEM or 5-fluoracil, which can be combined with radiotherapy. However, there are no conclusive data on a greater efficacy of chemo-radiotherapy against chemotherapy in this type of situation (127). Finally, for metastatic aggressive tumors, the goal of chemotherapy is mainly palliative, and it has been shown that GEM is more advantageous than 5-fluoracil in pain control, survival and quality of life (132). Multiple drugs have been evaluated in combination with GEM, but none of them have shown an additive significant benefit. The only drug that has shown a small but significant improvement when combined with GEM is erlotinib, an inhibitor of EGFR (Epidermal Growth Factor Receptor) (127).

Summarizing, it has been proved that the only alternative for cure or long-term survival for patients presenting pancreatic ductal adenocarcinoma is the surgical resection (126), but nevertheless, the 5-years survival rate is still too low. Identification of risk factors and early diagnosis improvements are mandatory in order to reduce the high death rates related to this disease (133).

The American Cancer Society divides the pancreatic cancer risk factors in two types: the modifiable ones, such as tobacco, overweight, diabetes, chronic pancreatitis and workplace exposure to certain chemicals, and the non-modifiable ones: age, gender, race, family history or chronic pancreatitis due to a gene change (125). For example, one important risk factor, chronic pancreatitis, has been studied in a recent meta-analysis (134), showing that the relative risk for pancreatic ductal adenocarcinoma in chronic pancreatitis is 13.3%, and it is even higher in hereditary pancreatitis. This suggests that patients with chronic pancreatitis, especially the genetic one, are a higher risk population for this type of cancer and could be diagnosed before (126). Other non-modifiable risk factors have been deeply analyzed, such as age or sex: an increased incidence of pancreatic cancer is noted with advanced age, as 60% of the patients are older than 65 years old, and men are 30% more likely to develop this cancer than woman, with Caucasians being less affected by this disease than Africans Americans (135).

It is important to mention that some genetic mutations, such as *PALB2, BRCA2*, or *P16*, have recently been identified as potential risk factors for pancreatic ductal adenocarcinoma, due to the increased incidence of this disease on those cases (136). In fact, pancreatic ductal adenocarcinoma is the result of the accumulation of successive mutations that have their origin in the pancreatic ductal epithelium (137). This epithelium progresses from normal to successive degrees of intraepithelial neoplasia, becoming finally an invasive carcinoma. At the same time, there are genetic mutations including the activation of the KRAS (90% of cases) and inactivation of tumor suppressor genes: *CDKN2A* (95% of cases), *P16* (95%), *P53* (50–75%), *DPC4* (55%) (127). Despite of this knowledge, the genetic basis of pancreatic cancer is still a very complex area of study, and it presents a lot of heterogeneity: some estimations show that this type of cancer can present an average of 63 genetic mutations (138).

These recent results about the implication of some genetic mutations in pancreatic ductal adenocarcinoma show the importance of finding reliable blood markers, in order to use them as preventive and/or prognosis biomarkers for this cancer. The actual absence of those reduces the potential effectiveness of a screening strategy in potential high-risk patients, even if there are increasing researches about this topic (126). Finally, it is important to mention that some patients with a large or significant family history of pancreatic ductal adenocarcinoma undergo close observation with abdominal magnetic resonance imaging in order to anticipate the diagnosis in case of a possible hereditary disease (139).

Unfortunately, even if there has been a large improvement on therapies, diagnosis procedures and study of the risk factors, pancreatic cancer does not have an actual therapy that significantly alters the course of pancreatic cancer, caused mainly by the difficulties to achieve an early diagnosis for this non-specific symptom disease (126). It is mandatory to continue researching about prevention, risk factors, possible prognosis or diagnosis biomarkers and combined/new therapies in order to reduce the high mortality rate caused by pancreatic ductal adenocarcinoma.

## Circadian Clock, Pancreatic Cancer, and Therapy

Few studies have shown specifically the influence of circadian clock genes on the biology of pancreatic cancer development and on the efficacy of the actual therapies used for the clinical management of the disease (140–143). Some of them report the effect of disruption of central clock on cancer growth in animal models. Disruption of SNC in mice induces downregulation of p53 and overexpression of c-Myc (144) and accelerates the growth of implanted human tumor cells from both osteosarcoma and pancreatic adenocarcinoma (145, 146). Circadian clock genes manipulation through controlled meal time in mice carrying small pieces of human pancreatic adenocarcinoma results in cancer grown inhibition by 40% vs. mice fed *ad libitum* (147).

At present, most research in relation to pancreatic cancer is focused on determining which genes are involved in the development and progression of this type of cancer, as well as to solve therapy resistance. Interestingly, circadian clock genes are implicated in both areas of cancer biology. A custom pancreas gene enriched microarray, the Pittsburgh Pancreas Gene Enriched ARray-PittPEAR, analyzed 5,763 genes, 264 of which were differentially expressed in pancreatic cancer vs. normal tissue (148). In this study were identified 30 human genes related to one of seven fly circadian genes. It was observed that one of the four human cytochrome P450-related genes, *PER1* and *DEC1*, and downstream effectors, such as ubiquitin specific protease 30 were significantly under-expressed in pancreatic cancer (148, 149). A most completed retrospective study using an integrated approach joining genomic, transcriptomic and clinical data demonstrated that the increased expression of *ARNTL2* and *NR1D1* had prognostic values for poorer survival outcomes, indicating that these genes could be prioritized as new therapeutic targets. Additionally, increase in tumor promoting properties resulted from circadian dysregulation is exacerbated by hypoxia. Therefore, the clock-hypoxia model may be used for delineation of patients to support adjuvant therapy with hypoxia-reducing drugs in combination with mainstream chemotherapy and radiotherapy (150).

Prospective analysis of circadian genes expression in patients with pancreatic ductal adenocarcinoma revealed lower expression of *PER1, PER2, PER3, CRY1, CRY2, TIPIN, TIM, CK1E, BMAL-ARNTL*, and *CLOCK* in cancer tissues compared to their matched adjacent tissue. It was also found a significant association between low expression levels of circadian genes and reduced survival (142). Specifically, high expression of *PER2* correlates with lower mortality (151). *BMAL1* overexpression significantly inhibited cell proliferation and invasion, and induced G2/M cell cycle arrest, whereas *BMAL1* knockdown promoted pancreatic cancer growth *in vitro*, probably by directly binding to the p53 gene promoter and thereby transcriptionally activating the downstream tumor suppressor pathway in a p53-dependent manner (152). In fact, immunohistochemistry analysis of Bmal1 in tumor tissues from 87 patients with pancreatic ductal adenocarcinoma showed lower levels of this protein compared with adjacent non-tumor tissues and low Bmal1 expression was associated with tumor progression and poor prognosis (143). In addition, high expression of *CRY2* and low expression of *DEC1* were associated with favorable prognosis in pancreatic cancer patients (153). Further, overexpression of mouse *Period2* gene (*mPer2*) in human pancreatic cancer cells reduced cellular proliferation and induced apoptotic cell death (141). Contrary to this, other authors found higher expression of *PER1* in pancreatic tumors vs. normal tissues (154). This gene acts as an intermediate in the inhibitory effects of TNFα (155) and its down-regulation increased apoptosis *in vitro* (154).

Currently there are few published studies on polymorphisms and pancreatic cancer (20). Cotterchio et al. found an association between the SNP rs12913421 in RORα and pancreatic cancer, although the significance was very weak (156).

Actually, the most effective treatment in pancreatic cancer is surgery resection together with neoadjuvant chemotherapy, as mentioned before. This scheme of treatment can include: GEM mono-therapy; oxaliplatin, irinotecan, fluorouracil, and leucovorin (FOLFIRINOX); GEM, docetaxel, capecitabine (GTX); GEM along with cisplatin; and the nanoparticle abraxane or albumin-bound (nab) paclitaxel along with GEM. The objective of GEM treatment is to inhibit DNA replication and, therefore, tumor growth but a high percentage of patients showed resistance to this treatment (157). Nowadays, different ways of treatment are being investigated as non-coding RNA, nanoparticles and liposome drugs, immunotherapy; chemoresistance related signaling pathway antagonists, molecular therapy and specific antibiotics to bacterial drug-activated enzyme (157, 158). Recently, it was reported that the molecular clockwork within malignant human pancreatic epithelium disruption and resistance to GEM is mediated by miR-135b-induced *BMAL1* repression. Moreover, authors found that YY1 transcriptionally activated miR-135b and formed a “miR-135b-*BMAL1*-YY1” loop, which has significant predictive and prognostic value for patients with pancreatic cancer (159). *PER*2 has a critical role in controlling the malignancy of cancers and also showed a mechanism regulating the resistance of oncogene-transformed *PER2*^m/m^ cells against the cytotoxicity of chemotherapeutic drugs. *PER2* mutated cannot be translocated to the nucleus to bind to histone deacetylase (*HDAC*) preventing the deacetylation of H3K9, this causes an increase in the expression of aldehyde dehydrogenase 3a1 (*ALDH3A1*) that blocks ROS generated by chemotherapeutic agents (160). Reduction of Bcl-XL expression levels induced by overexpression of *mPER2* may influence the sensitivity to anticancer agent cisplatin (CDDP) in human pancreatic cancer cells enhancing tumor death (141).

From an epigenetic point of view, drugs are developed to reverse the global epigenetic alterations that occur in cancer. Inhibitors of DNA methylation can be used in single or combined therapy to synergistically induce apoptosis of tumor cells and reverse resistance to therapy in some types of cancer, including pancreatic cancer. Several inhibitors of HDAC are currently in clinical trials, showing less toxicity and adverse effects than conventional cancer therapies (33, 161). Although the link between the circadian epigenome and cancer remains unclear, it has been used as a clinical approach in the treatment of cancer *in vitro* and *in vivo* models of the disease (33). Therefore, they could be used as treatments for pancreatic cancer, given that numerous studies have shown the presence of alterations in the expression of HDACS in this type of cancer.

High HDAC4 and HDAC7 expression were significantly associated with the presence of adenocarcinomas of the pancreas (162, 163). Suberoylanilide hydroxamic acid (SAHA) is an irreversible pan HDAC inhibitor, which was approved for the treatment of cutaneous T-cell lymphoma (164). This drug inhibits class I and II HDACs with higher IC50 for HDAC 4, 7, and 9 (165). SAHA also appears to be a promising therapeutic approach alone or in combination with sorafenib for the treatment of HCC (166). MC1568 is another HDAC7 inhibitor that improves insulin secretion from type 2 diabetes patients and rescues β-cell dysfunction caused by HDAC7 upregulation (167). These drugs could be assayed for the treatment of pancreatic cancer.

SIRT1 is a very important regulator and potential therapeutic target in pancreatic carcinogenesis and in advanced pancreatic cancer. Its overexpression is associated with metastasis of pancreatic ductal adenocarcinoma and promotes migration and growth of pancreatic cancer cells (168). This could be related with the ability of SIRT1 to reduce E-cadherin transcription activity increasing cell EMT (169). Other sirtuins, such as SIRT3 and SIRT7 also possess tumor suppressor properties in pancreatic cancer. In addition, SIRT3 may represent a predictive biomarker of response to chemotherapy (170).

SIRT1 regulates a large number of proteins often functionally implicated in tumor development and progression (171, 172). SIRT1 and deleted in breast cancer 1 (DBC1) co-express in the nuclei of exocrine pancreas. In acinar-to-ductal metaplasia (ADM), Dbc1 remains in the nucleus whereas SIRT1 underwent a transient nuclear-to-cytoplasmic shuttling and regulates acinar cell differentiation. Furthermore, inhibition of SIRT1 is effective in suppression of ADM and in reducing cell viability in established pancreatic tumors, where it acts as an independent prognostic factor for survival in patients (173). The sensitivity of pancreatic cancer cells to the Sirt1/2 inhibitor Tenovin-6 correlates with the levels of SIRT1/Dbc1 suggesting that Dbc1 can be a biomarker for those pancreatic tumors that could benefit from SIRT1-inhibitory drugs (174). Capsaicin induces apoptosis in pancreatic cancer *in vitro* leading to the acetylation of FOXO-1 through activation of CBP and inhibition of SIRT-1 (175). Plumbagin (PLB), an active naphthoquinone compound, promotes cell cycle arrest and autophagy but inhibits EMT phenotype in pancreatic cancer cells with the involvement of SIRT1 (176). Similarly, alisertib (ALS), a potent and selective Aurora kinase A inhibitor, induces cell cycle arrest and autophagy and suppresses EMT involving PI3K/Akt/mTOR and SIRT1-mediated signaling pathways in human pancreatic cancer cells (177). Contrary to the above results, the SIRT1-activating compounds SRT1720, SRT1460, and SRT3025 inhibit cell growth and survival of pancreatic cancer cells and enhance the sensitivity of pancreatic cells to GEM and paclitaxel (178).

Combined therapy of GEM with inhibition of SIRT1, improve efficacy and survival time in a pancreatic cancer xenogeneic mice model, compared with single inhibition of SIRT1, or single GEM therapy (179), probably due to the implication of SIRT1 in chemo-sensitivity of pancreatic cancer cells (180). SIRT1 seems to be involved in the adaptive response of pancreatic cancer cells to chemotherapy-induced DNA damage stress (181). The SIRT1 inhibitor 6-chloro-2,3,4,9-tetrahydro-1 H-Carbazole-1-carboxamide (EX527) enhanced sensitivity of pancreatic cancer cells to GEM treatment through increased apoptosis (182). In line with this, two MDM2 inhibitors (MI-319 and MI-219) synergistically augmented anti-tumor effects of therapeutic drug GEM in pancreatic cancer *in vitro* and *in vivo* experimental models trough the reactivation of p53 pathway and targeting SIRT1 and Ku70 (183). In addition, resistance to TRAIL-induced apoptosis seems to be mediated by SIRT1 and HDAC3 (184).

There is intense research to identify nontoxic but high potency inhibitors of SIRT1 (185). Melatonin or (N -acetyl-5-methoxytryptamine) is a phylogenetically well-preserved indoleamine synthesized from tryptophan in the pineal gland and in other many tissues of human body (186). Anti-tumor effects of this indoleamine have been described *in vitro* and *in vivo* models of this disease as single or combined therapy (187–189). In the case of combined therapy, it has been shown to increase the efficacy and decrease the toxicity of the current treatments of cancer (190). Many mechanisms have been proposed to explain the effects of melatonin in cancer. As an important regulator of circadian rhythms, it acts on different types of cancer, including pancreatic cancer (189, 191). Direct and indirect approaches showed a dual relationship between melatonin and SIRT1 in normal and tumor cells. In several cancer models, melatonin inhibits SIRT1 activity. However, there are no direct evidences showing a role for melatonin in regulating cancer cell growth through SIRT1/circadian clock axis (192). Melatonin can also be found in mitochondria of mammalian cells were it seems to activate SIRT3 leading to decreased ROS production, among other effects. Given the relationship between redox homeostasis of the cells and the circadian clock, this could be an indirect mechanism by which melatonin could regulate the circadian clock in cancer cells through sirtuins, although it has not been yet studied (193).

Given the importance of SIRT1 in pancreatic cancer regulation, it was expected that NAD synthesis and degradation had an important role in tumor cell metabolism and growth as well (194). Pharmacologic and genetic targeting of *NAMPT*, the key enzyme in the NAD salvage synthesis pathway, inhibits cell growth and survival of pancreatic cancer cells. The responsiveness to *NAMPT* inhibition is modulated by the expression of CD38. However, neither SIRT1 nor PARP-1 (another NAD+ dependent enzyme) play a significant role on the effect of *NAMPT* inhibition on pancreatic cancer cells (194).

Other pharmacological approaches can be directed to some miRNAs responsible for the regulation of SIRT1 expression. MiR-34a is known to be downregulated in the majority of pancreatic cancers. It is a component of the p53 transcriptional network and regulates cancer stem cell survival. Its restitution using a systemic nanovector inhibits pancreatic cancer growth in mice and decreases SIRT1 expression (195). Interestingly, demethylating agent 5-Aza-2'-deoxycytidine (5-Aza-dC) and HDAC inhibitor SAHA can also restore miR-34a expression in human pancreatic cancer stem cells (CSCs) and in human pancreatic cancer cell lines and strongly inhibit the cell proliferation, cell cycle progression, self-renewal, epithelial to mesenchymal transition (EMT) and invasion (196). Ectopic expression of miR-217 inhibited TGF-β1-induced EMT by downregulating SIRT1 in chronic pancreatitis and pancreatic cancer (197), while miR-494 inhibited the proliferation, invasion and chemoresistance of pancreatic cancer by regulating SIRT1 and c-Myc (198). MiR-601 also inhibits SIRT1 and its expression is significantly lower in cancer samples, especially in metastatic compared to non-metastatic pancreatic cancer tissues (199).

Autophagy is the dynamic process by which unnecessary or dysfunctional cytosolic proteins and organelles are degraded in order to maintain cell homeostasis. It is activated under extracellular (starvation, hypoxia and infection) and intracellular (accumulation of damaged molecules and high bioenergetics demand) stress conditions (200). Nutrient limitation or starvation is a frequent feature of the cellular microenvironment in the core of solid tumors. The predominant role of autophagy in cancer cells is to confer stress tolerance, which serves to maintain tumor cell survival (201) by altering metabolic conditions (200).

Autophagy has been associated to pancreatic cancer and activated autophagy supports tumor growth and plays a role in the lack of effect of current treatments (202). The effects of autophagy modulation in this cancer seem to depend on both tumor specific properties and the chemotherapy schedule (203). However, the mediators that regulate the crosstalk between autophagy and apoptotic death in cells exposed to extreme nutrient starvation in pancreatic cancer are not fully understood. Recent studies suggested that glucose starvation induces progressive autophagy trough GPx1 degradation and inhibition of glycolysis activation and subsequent activation of ROS/AMPK signaling (204). Other studies showed the implication of mTORC1 in the induction of autophagy by starvation in several types of cancer, including pancreatic cancer. MiT/TFE transcription factors, master regulators of lysosomal and melanosomal biogenesis and autophagy, control mTORC1 lysosomal recruitment and activity by directly regulating the expression of RagD (205). Wong and coworkers demonstrated the induction of autophagy by serum plus amino acids starvation in pancreatic ductal adenocarcinoma cells. Suppression of mTORC1 activity by starvation but not mTORC1 inhibitors triggers dissociation of PP2A from its inhibitor Alpha4 and ULK1 dephosphorylation at S637 (206). On these conditions, a signaling cascade involving AMPK and SIRT1 displaces chromatin-bound BRD4, inducing autophagy gene activation and cell survival (207). On the other hand, miR-138-5p suppresses autophagy by directly targeting SIRT1 expression and inhibits mTOR dephosphorylation under serum starvation-induced autophagy in pancreatic cancer (208). These mechanisms could be under the control of circadian clock trough SIRT1. In fact, serum starvation of pancreatic cancer *in vitro* induces increased SIRT1 activity and decreased levels and temporal patterns of expression of circadian clock genes *in vitro* (151).

Taking together, all this results showed the feasibility of the direct or indirect handling of circadian clock as an approach to treat pancreatic cancer. Optimizing the time of drug administration may also offer an improvement of drug efficacy and safety without neither increasing drug doses nor changing drug types. Currently, several randomized controlled trials and clinical practices highlight the validity of circadian-based treatments (209). However, the absence of a systematic computation for circadian timing in cancer therapies makes it a challenge (210). In addition, a comprehensive characterization of clock genes and their clinical relevance in cancer is also necessary. In line with this, Ye and coworkers analyzed genomic profiling and clinical data from 32 cancer types, including pancreatic cancer, from The Cancer Genome Atlas (TCGA) (211), the Cancer Therapeutics Response Portal (CTRP) (212), and Genomics of Drug Sensitivity in Cancer (GDSC) databases (213). They found alterations of clock genes at transcriptional, genetic and epigenetic levels and identified ARNTL2, NR1D1, and NPAS2 as probable oncogenes and PERs, CRYs, RORs as gene suppressors of tumors. Transcriptional dysregulation of clock genes is strongly associated with patient survival, tumor stage, and subtype. Further, they identified the effect of dysregulated clock genes on different signaling pathways and the potential therapeutic effects of clock genes in cancer chronotherapy. Their results highlight the potential therapeutic use of the right time schedule of a treatment in cancer as well as the need to personalize chronotherapy. However, more research is necessary in this field because the results concerning drugs effect were obtained from cells and the time at which the specimen from patients were obtained is unknown (59).

## Concluding Remarks

**Figure 1:**
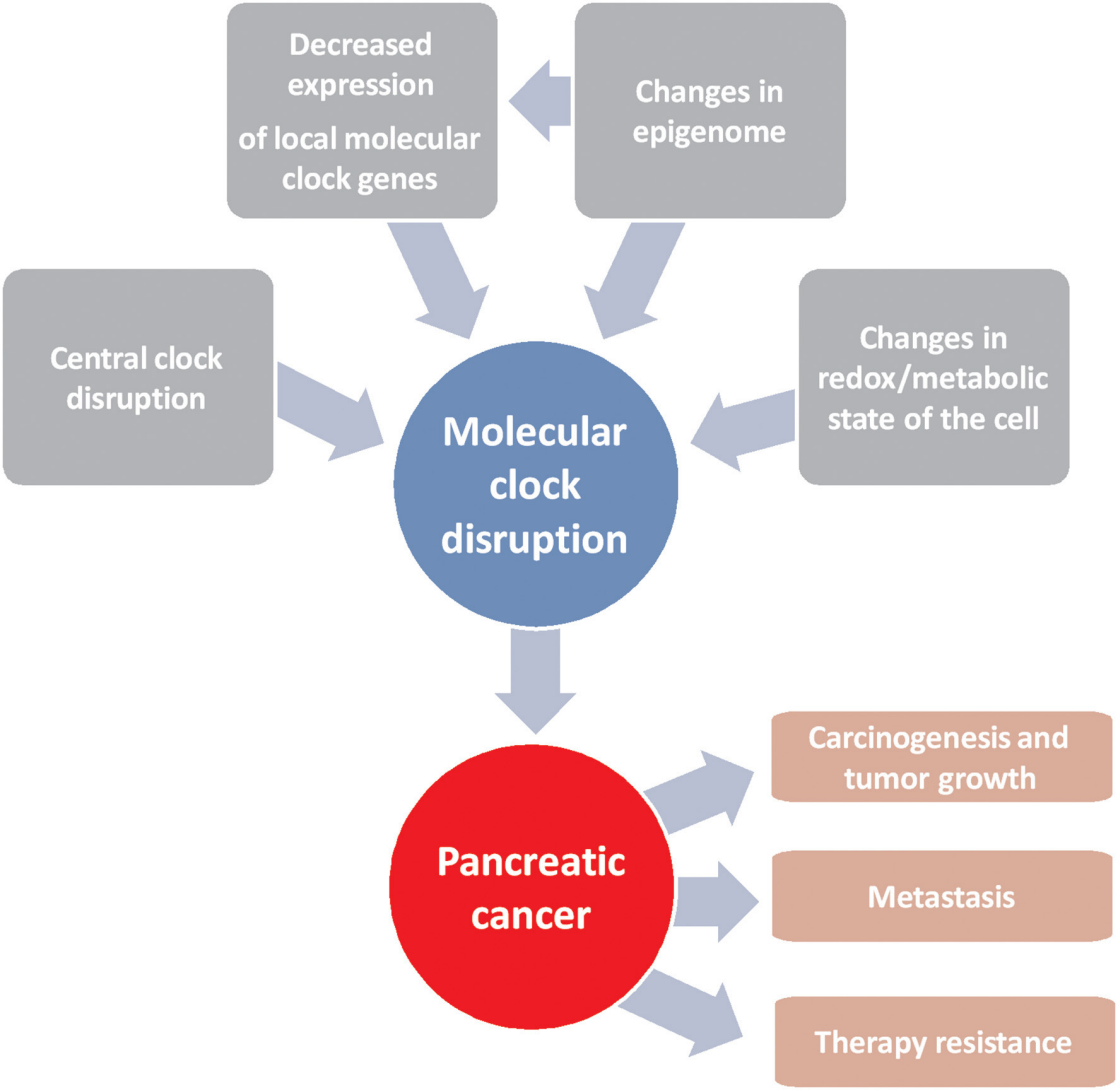
Schematic representation of factors driving alterations in the molecular clock that lead to the development of pancreatic cancer and resistance to chemotherapy.

Pancreatic cancer is one of the most deadly types of cancer due to the lack of early diagnosis and to the lack of specific molecular targets for its treatment, despite the efforts made in recent decades in this regard. The use of personalized medicine is crucial, as clinical heterogeneity is a hallmark of this disease. Molecular clock disruption through several ways leads to carcinogenesis, tumor growth and therapy resistance (Figure 1). In this sense, components of molecular circadian clock are emerging tools that can be used as prognostic factors for effectivity of commonly used chemotherapies and for the development of new treatments. Despite the results obtained in this field of research, the clinical trials necessary to take advantage of them have not been launched. On the contrary, in relation to prognosis, controversial results obtained from patients make necessary more research before to use circadian molecular clock components as biological markers of disease prognosis and outcome.

## Author Contributions

JL and ÁC conceived the project. JL, JP-P, MG-C, JE-F, SM-S, and SR-A wrote the paper. JL and JP-P contributed to the revision of literature. All authors have read, corrected, and approved the manuscript.

## Conflict of Interest

The authors declare that the research was conducted in the absence of any commercial or financial relationships that could be construed as a potential conflict of interest.

## Footnotes

^1^page, A.C.S.w. *American Cancer Society web page* [Online]. Available online at: https://www.cancer.org/cancer/pancreatic-cancer.html [Accessed].
